# Lightweight Indoor Multi-Object Tracking in Overlapping FOV Multi-Camera Environments

**DOI:** 10.3390/s22145267

**Published:** 2022-07-14

**Authors:** Jungik Jang, Minjae Seon, Jaehyuk Choi

**Affiliations:** School of Computing, Gachon University, 1342 Seongnam-daero, Sujeong-gu, Seongnam-si 13120, Korea; jji4449@gachon.ac.kr (J.J.); ddol0225@gachon.ac.kr (M.S.)

**Keywords:** multi-camera tracking, multi-target tracking, global tracklet matching, Dynamic Time Warping, KLT algorithm

## Abstract

Multi-Target Multi-Camera Tracking (MTMCT), which aims to track multiple targets within a multi-camera network, has recently attracted considerable attention due to its wide range of applications. The main challenge of MTMCT is to match local tracklets (i.e., sub-trajectories) obtained by different cameras and to combine them into global trajectories across the multi-camera network. This paper addresses the cross-camera tracklet matching problem in scenarios with partially overlapping fields of view (FOVs), such as indoor multi-camera environments. We present a new lightweight matching method for the MTMC task that employs similarity analysis for location features. The proposed approach comprises two steps: (i) extracting the motion information of targets based on a ground projection method and (ii) matching the tracklets using similarity analysis based on the Dynamic Time Warping (DTW) algorithm. We use a Kanade–Lucas–Tomasi (KLT) algorithm-based frame-skipping method to reduce the computational overhead in object detection and to produce a smooth estimate of the target’s local tracklets. To improve matching accuracy, we also investigate three different location features to determine the most appropriate feature for similarity analysis. The effectiveness of the proposed method has been evaluated through real experiments, demonstrating its ability to accurately match local tracklets.

## 1. Introduction

Multi-Target Multi-Camera Tracking (MTMCT) has recently received considerable attention due to the growing demand for intelligent monitoring and surveillance systems. It aims to track multiple interested targets and infer a complete trajectory for each target across a multiple-camera network [1]. MTMCT can be applied to various tasks such as video surveillance [1,2,3], city-scale traffic management [4], smart buildings [5,6], and in-store customer analysis [7].

Owing to the rapid development of object detection techniques [8,9,10,11], most state-of-the-art MTMCT methods employ a two-phase pipeline, namely detection-and-tracking  [12], to focus on the tracking functionality. In the first phase, they detect targets using a modern object detector and generate sets of local trajectories for each detected target within a single camera. To track targets within the entire multi-camera network, the cross-camera tracklet matching is performed in the next phase, which matches local tracklets across all the cameras to generate a complete trajectory for each target [1].

However, it is a challenging task, as these methods are inherently prone to camera field-of-view (FOV) issues, such as occlusion, i.e., the blind areas of camera views, and/or a significant change in the visual appearance of moving targets. As a result, the trajectory of each target generated within each camera is easily divided into multiple local tracklets, i.e., sub-trajectories. In addition, the tracker can generate incorrect duplicate local tracklets for the same target, which makes the cross-camera tracklet matching problem even more challenging. The cross-camera global tracklet matching task exhibits high computational complexity, and thus, it may not be available for real-time applications.

In this paper, we tackle the cross-camera tracklet matching problem in MTMCT, which focuses on the multi-camera association of local trajectories from each camera in overlapping FOV multi-camera environments. To this end, we present an MTMC framework that takes videos from multiple cameras and generates global trajectories for targets using light-weight similarity analysis based on the Dynamic Time Warping (DTW) algorithm. Our system consists of three major components: (1) Multi-Object Tracker (Section 3.2), (2) Ground Projector (Section 3.3), and (3) Global Trajectory Mapper (Section 3.4). As we will elaborate in Section 3, the first component, Multi-Object Tracker, considers an input video clip from each camera in the network and generates a set of local image information such as target ID, bounding boxes, and locations. Then, Ground Projector processes local tracklets to generate features suitable for similarity analysis by projecting the tracklets to the ground or bird’s-eye views. Moreover, we alleviate the inborn position error caused by camera distortion by exploiting the moving direction information of the target, instead of the absolute position of the target, for similarity analysis. In order to effectively predict the moving direction of the target and reduce the computational cost, we utilize a Kanade–Lucas–Tomasi (KLT) algorithm-based  [13,14] frame-skipping method that uses only one frame per n-frames instead of using all the frame information. In addition, we have created the attribute sequence of each target using three different features, namely, scalar, vector, and unit vector, to perform similarity analysis. Finally, Global Trajectory Mapper analyzes similarity based on the DTW algorithm and generates a complete global trajectory and ID for each target across the multiple cameras. We demonstrate the accuracy of our approach through extensive real experiments, where we constructed FOV environments by installing multiple cameras (up to four cameras) in various places, including classrooms and daycare centers. The evaluation results have shown that our approach is highly accurate in matching local tracklets and tolerant to noises with a low computational overhead.

We summarize the main contributions of this study as follows:

(1) Introduction of a new MTMC framework for overlapping FOV multi-camera networks (Section 3.1).
(2) Proposal of a lightweight global tracklet matching algorithm based on DTW similarity analysis (Section 3.4).
(3) Investigation of several movement features of targets to generate sequence sets for similarity analysis (Section 3.3).
(4) Implementation and evaluation of a prototype of the proposed framework with extensive real experiments (Section 4).

The rest of the paper is organized as follows: Section 2 discusses the related work and illustrates the preliminaries. Section 3 details the proposed framework and its main components. Section 4 demonstrates the evaluation results. Section 5 concludes the study.

## 2. Related Work

### 2.1. Multi-Target Multi-Camera Tracking (MTMCT)

The MTMCT task aims to infer the perfect path for each target in multi-camera environments. It usually comprises two steps: (1) generating local tracklets of all targets within each camera, and (2) matching the local tracklets for the same global target across a multi-camera network. In recent years, studies on the MTMCT task based on various approaches have been actively conducted.

Fleuret et al. [15] proposed a solution that utilizes a probabilistic occupancy map (POM) to approximate the probabilities of occupancy and combines it with the usual color and motion attributes. Berclaz et al. [16] optimized the MTMCT task by employing the POM and K-Shortest Path (KSP) algorithm. Hu et al. [17] and Eshel and Moses [18] presented a matching algorithm for the same target across multiple cameras using the homography correspondence. Similarly, the proposed method employs homography; however, it uses the DTW algorithm to match global IDs. Hou et al. [19] presented a new approach focusing on local neighboring data matching using a Locality Aware Appearance Metric (LAAM) composed of a metric network. Bredereck et al. [20] matched local tracklets of all cameras using the greedy matching association method. As an example of solving the MTMCT task using hierarchical clustering, Zhang et al. [21] proposed an approach that uses the distance matrix between averaged Re-ID features and applies re-ranking [22] to cluster local tracklets. Jiang et al. [23] solved the problem of trajectory association under orientation variations and occlusions, and they improved the matching efficiency using camera topology. Xu et al. [24] presented an approach using a hierarchical composition model for MTMCT. They re-formulated MTMCT as a composition structure optimization problem. He et al. [1] obtained the tracklet-TID assignment matrix with the Restricted Non-negative Matrix Factorization (RNMF) algorithm and used it to match the tracklet to the target ID (TRACTA). You et al. [25] used Optical-based Pose Association (OPA) for MTMCT and solved the occlusion problem using local pose matching. In addition, the distance problem caused by fast motion was reduced by applying optical flow. Wu et al. [26] proposed a three-step cooperative tracking method to track people in a multi-camera environment through tracking token transfer. Zhang et al. [27] proposed an online (real-time) tracking framework, and they improve the cross-camera person recall performance through appearance and spatial–temporal features. The MTMCT task has been widely used to target vehicles and humans. Hsu et al. [28] proposed the vehicle MTMCT framework, Trajectory-based Camera Link Model (TCLM), through which spatial–temporal information is obtained and MTMCT performance is improved by reducing the Re-ID candidate search process.

### 2.2. Multi-Object Tracking (MOT)

Multi-Object Tracking (MOT), which is the first step among the process steps of MTMCT described above, can be viewed as a problem of tracking multiple objects in a single camera. MOT aims to estimate and associate a bounding box and ID for a number of objects appearing in an image. As our study utilizes the object information (tracklet) of local image data, which are generated by MOT, the accuracy of the proposed method relies on the MOT performance. The MOT task can be divided into two methods, namely detection-by-tracking and tracking-by-detection, depending on how detection results are used. The tracking-by-detection method has recently attracted attention with the advent of high-performance object detection models. Moreover, it can be categorized into two approaches: (1) a batch tracking method in which data are correlated using the entire data frame information and (2) an online tracking method in which data are correlated using past and current frame information. SORT was proposed by Bewley et al. [8]; it is a popular method that uses online tracking, predicts the tracklet position for a new frame using a Kalman filter [29] and correlates the data using a Hungarian algorithm [30]. DeepSORT [9] supplements the ID switching problem caused by occlusion, which is a problem in SORT, with Deep Appearance Descriptor, and it enables more accurate tracking with the cascated matching strategy. FastMOT [31] used in our study solves the bottleneck caused by the use of DeepSORT’s two-stage tracker by running the detector and feature extractor every specific frame. In addition, motion compensation makes it possible to track objects with a moving camera. Most MOT-related studies deal with outdoor tracking such as video surveillance and autonomous driving; however, there is a clear difference from indoor environments. Therefore, Liu et al. [32] presented the depth-enhanced tracking-by-detection (DET) framework optimized for the indoor environment where occlusion frequently occurs. ByteTrack [11], which has recently achieved state-of-the-art results in the field of MOT, dramatically improves performance by associating a detection box with an object with a low detection score. ByteTrack achieves the highest performance, but we wanted to take advantage of FastMOT’s skip function, which enhances the frame processing speed. In addition, there are various MOT methods [10,33,34,35,36,37,38,39,40,41,42,43,44,45]. In the experimental stage, the accuracy of global ID matching is measured by changing the skip parameter value. In the proposed framework, the MOT model can be replaced by other models.

## 3. System Design

This section presents an overview of the proposed system and its main components.

### 3.1. System Architecture

**Figure 1:**
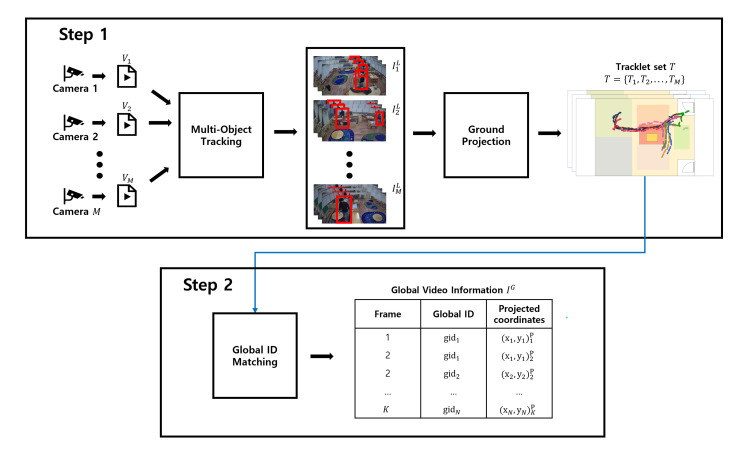
System Architecture.

Figure 1 illustrates the high-level overview of our proposed system. We consider a multi-camera network comprising *M* cameras. The proposed system takes *M* input video clips from each camera and generates a complete trajectory for each target across the *M* camera network. It consists of three components: (1) a Multi-Object Tracker (Section 3.2), (2) Ground Projector (Section 3.3), and (3) Global Trajectory Mapper (Section 3.4); these components perform two steps: (i) local tracklet generation and (ii) complete global trajectory generation. As shown in Figure 1, the first two components handle the first step, and the latter one performs the second step. We explain each component and phase in detail in the following sub-sections.

### 3.2. Multi-Object Tracker

Given input video $V_{i}$ from the *i*-th camera (i=1,…,M), *Multi-Object Tracker* detects a set of targets and generates their local tracklet information. As mentioned in the previous section, we rely on the state-of-the-art modern multiple object tracker for this task. To detect the targets, i.e., people, in the field-of-view (FOV) and their bounding boxes, we have employed FastMOT [31], which significantly accelerates the object tacking system to run in real time.

As the output of input video $V_{i}$ (i=1,…,M), Multi-Object Tracker generates a set of frame-by-frame local object detection information, $I_{i}^{L}$, which is given by

$$I_{i}^{L}=\left\{I_{i,1}^{L},I_{i,2}^{L},\ldots ,I_{i,K}^{L}\right\},$$

where *K* is the number of frames in video $V_{i}$ and $I_{i,k}^{L}$ denotes a set of local image tuples for each target detected in the *k*-th frame (k=1,…,K). Here, each video $V_{i}$ may have a different value of *K* and should be denoted as $K_{i}$. However, we omit the subscript *i* to simplify the notation throughout the paper.

Let $\Pi_{i}$ denote a set of targets’ local IDs (LIDs) observed in video $V_{i}$ and $\pi_{i,k}$ denote a set of LIDs observed in the *k*-th frame in $V_{i}$. Then, we can obtain

(1) $$\Pi_{i}=\overset{K}{\underset{k=1}{⋃}}\pi_{i,k}.$$

A set of local image information $I_{i,k}^{L}$ for the *k*-th frame (k=1,…,K) is composed of a series of tuples $u_{i,k}\left(LID\right)$ with three attributes: (1) the frame number *k*, (2) the target’s local ID, namely LID, and (3) the local foot coordinates (bottom center of the bounding box, which is often assumed to be on the ground):

(2) $$I_{i,k}^{L}=\left\{u_{i,k}\left(LID\right):=\left(k,LID,{\left(x,y\right)}^{L}\right)|LID\in \pi_{i,k}\right\},$$

where the coordinate ${\left(x,y\right)}^{L}$ is the reference location of target $ID^{L}$, which is the bottom center of its bounding box.

One of the challenges at this stage is that the object detection method demonstrates large temporal variations when it generates the bounding box of the target across the frame, where fluctuations may occur owing to motion blur, partial occlusion, change in poses, and other factors. This can cause short-term fluctuations in the derivation of local foot coordinates ${\left(x,y\right)}^{L}$ that act as short-term noise values.

To handle this issue, we use a simple yet effective strategy, known as Frame Skipping, which detects and tracks only the selected frames at a specific sampling period *s*. Frame Skipping predicts target positions using the KLT tracker without executing the detector and feature extractor for the frames between two selected frames. For a skipping period *s* and a certain selected *k*-th frame, $I_{i,k+1},I_{i,k+2},\ldots ,I_{i,k+s-1}$ are estimated by the KLT tracker. Frame Skipping alleviates the bottleneck of traditional MOT methods and enables real-time execution. In addition, we will show that the accuracy of the proposed matching algorithm is improved by removing noise in the calculation of the direction of each target (vector features), which will be described in Section 3.4.

### 3.3. Bird’s Eye View (BEV) Ground Projector and Feature Extractor

*Ground Projector* takes the set of local image information in $I^{L}=\left\{I_{1}^{L},I_{2}^{L},\ldots ,I_{M}^{L}\right\}$ and for each $I_{i}^{L}$ (i=1,…,M) produces the set of coordinates ${\left(x,y\right)}^{P}$ projected on the target coordination map by using a *homography matrix* *H*; homography is a transformation process (a 3 × 3 matrix) that maps the points in one image to the corresponding points in the other image [46].

To obtain the homography matrix $H_{i}\left(\in H\right)$ for $V_{i}$, eight point coordinates are needed, including four point coordinates in the image data and four corresponding points in the projection map. We have obtained reference points in the experiments by placing a rectangular grid carpet and by measuring edge positions offline.

**Figure 2:**
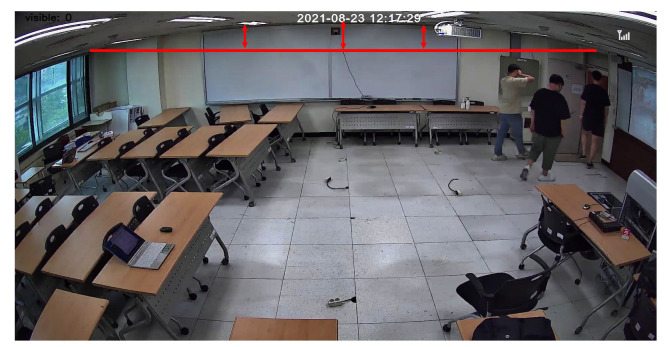
Barrel distortion commonly observed in commercial cameras.

In our experiments, we have observed a well-known camera distortion problem, i.e., barrel distortion [47], at the edges of frames (Figure 2). This distortion significantly affects on-the-ground projection, especially depending on the installation height and angle of the camera. This can cause a decrease in accuracy with regard to the performance of our similarity-based global ID-matching algorithm.

Therefore, camera calibration including distortion correction is required to obtain the accurate matrix *H* and improve the matching accuracy. The parameters derived for camera calibration can be used continuously once acquired; however, if the camera’s angle of view is changed, it must be derived again.

One key observation to address this challenge is that the error caused by camera distortion significantly affects the derivation of the targets’ coordinates, but it negligibly impacts on the derivation of the targets’ moving direction. Based on this observation, we adopt the approach of using the targets’ movement direction as the key feature for our global ID-matching algorithm instead of a cost calibration process. In particular, we investigate the vector-based features of moving targets for the global ID-matching algorithm, which will be described in Section 3.4.

**Figure 3:**
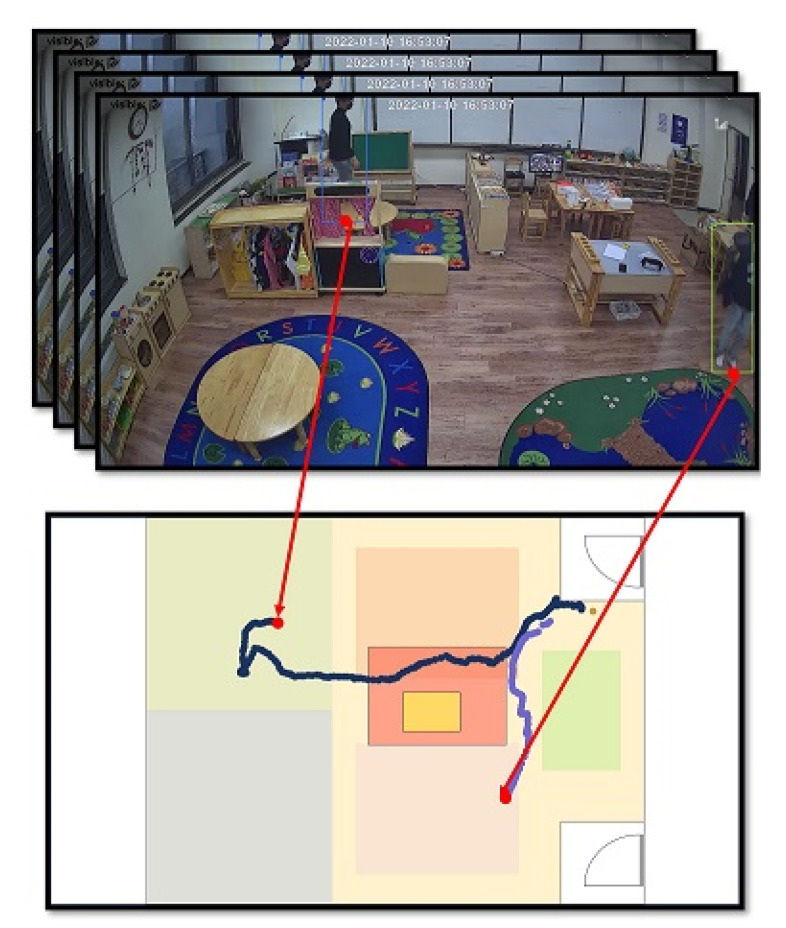
Local tracklet represented in the projection map (bird’s eye view).

Each local image information $I_{i,k}^{L}$ in Equation (2), which contains local coordinates ${\left(x,y\right)}^{L}$, is projected into the corresponding coordinates ${\left(x,y\right)}^{P}$ on the target map (Figure 3), which will be to generate a set of projected local image tuples $I_{i,k}^{P}$ for the *k*-th frame (k=1,…,K):

(3) $$I_{i,k}^{P}=\left\{u_{i,k}\left(LID\right):=\left(k,LID,{\left(x,y\right)}^{P}\right)|LID\in \pi_{i,k}\right\}.$$

Then, for each target with local ID x $\in \Pi_{i}$, we can construct the local tracklet $T_{i,x}^{L}$ as a set of tuples over the *k*-th frame (k=1,2,…,K) in $V_{i}$:

(4) $$T_{i,x}^{L}=\overset{K}{\underset{k=1}{⋃}}\left\{u_{i,k}\left(x\right)|x\in \pi_{k,i}\right\}.$$

Let $T_{i}^{L}$ denote a set of local trackets for $V_{i}$ extracted from the *i*-th camera, which is given by

(5) $$T_{i}^{L}=\left\{T_{i,1}^{L},T_{i,2}^{L},\ldots ,T_{i,N}^{L}\right\},$$

where *N* is the total number of targets observed across all the cameras, given by $N=|{⋃}_{i=1}^{M}\Pi_{i}|$. Here, the number of targets in $V_{i}$ may be less than *N* because a few targets may never appear in camera *i*.

For example, when the *p*-th local ID is detected in the image data with the frame range [1,4,…,1024], $T_{i,p}$ for $V_{i}$ is given as:

$$T_{i,p}=\left(\begin{array}{c} \left\{frame_{1},{local-id}_{p}^{L},{\left(x_{p,1},y_{p,1}\right)}^{P}\right\}\\ \left\{frame_{4},{local-id}_{p}^{L},{\left(x_{p,4},y_{p,4}\right)}^{P}\right\}\\ \ldots \\ \left\{frame_{1024},{local-id}_{p}^{L},{\left(x_{p,1024},y_{p,1024}\right)}^{P}\right\} \end{array}\right).$$

Figure 3 shows the local tracklet set *T* on the projection map. A set of *M* local tracklets generated across the multi-camera network is used as an input for the global ID-matching module.

### 3.4. Global ID Matching

Finally, given *M* sets of local tracklets $T_{i}^{L}$ (i=1,2,…,M) from *M* cameras, the global ID-matching component uses the DTW (Dynamic Time Warping) algorithm to perform similarity analysis. The DTW algorithm can measure the similarity of two sequences, and it has the advantage of being able to measure even if the lengths of input sequences are different from each other. We used the two-dimensional sequence set $S_{i,a}$ ($\forall a\in \Pi_{i}$) generated from the coordinate information of $T_{i,a}$ to measure the similarity.

The features used for the input sequence generation are (i) scalar, (ii) vector, and (iii) unit vector of each target’s movement information. In particular, to generate $S_{i,a}$ for target *a*, the coordinates ${\left(x,y\right)}_{k}^{P}\left(a\right)$ and ${\left(x,y\right)}_{k+1}^{P}\left(a\right)$ of two adjacent frames (i.e., *k*-th and k + 1-th frames) in tuples $u_{i,k}\left(x\right)$ and $u_{i,k+1}\left(x\right)$$\in T_{i,x}$ are used. The *k*-th element values of $S_{i,a}$ generated using the (i) scalar, (ii) vector, and (iii) unit vector are $s_{k}$, $v_{k}$, and $w_{k}$, respectively, which are given by:

(6) $$s_{k}=\left|{\left(x,y\right)}_{k+1}^{P}\left(a\right)-{\left(x,y\right)}_{k}^{P}\left(a\right)\right|,$$

(7) $$v_{k}={\left(x,y\right)}_{k+1}^{P}\left(a\right)-{\left(x,y\right)}_{k}^{P}\left(a\right),$$

(8) $$w_{k}=v_{k}/\left|v_{k}\right|.$$

Algorithm  1 can generate sequences using the aforementioned features. To compare the similarity between synchronized trajectories, we compute the sequence of each target’s local tracklet over the same period of time (frames).

**Algorithm 1:** Generation of Sequence Set $S_{i}$
**Input** **:**    Local tracklet set $T_{i}^{L}=\left\{T_{i,1}^{L},T_{i,2}^{L},\ldots ,T_{i,a}^{L},\ldots ,T_{i,N}^{L}\right\}$   for each target *a*($=1,2,\ldots ,N\;=\;|\Pi_{i}|$) generated from video $V_{i}$**Output** **:**    Vector sequence set $S_{i}^{L}=\left\{S_{i,1}^{L},S_{i,2}^{L},\ldots ,S_{i,N}^{L}\right\}$
1:
**for all** $T_{i,a}^{L}\in T_{i}^{L}$ **do**
2: **for all** $u_{i,k}(a)\in T_{i,a}^{L}$ **do**3:  Generate $v_{k}$ (or $s_{k}$, $w_{k}$) using Equation (7) (or Equation (6), Equation (8), respectively)4:  and add $v_{k}$ (or $s_{k}$, $w_{k}$) to $S_{i,a}$5: **end for**6:
**end for**



Given the sequence sets of each target, we use the DTW distance function $d_{DTW}\left(S_{i,a},S_{j,b}\right)$ for two different sequences from the *i*-th and *j*-cameras (*i* and *j*∈ {1, 2, …, *M*}, i≠j) to calculate the distance value *D*:

(9) $$D\left(S_{i,a},S_{j,b}\right)=d_{DTW}\left(S_{i,a},S_{j,b}\right),$$

where the lower the *D* value is, the higher the similarity will be.

We use Algorithm 2 to perform global ID matching by calculating a Tracklet Similarity Candidate List *R*. Under the overlapping FOV condition that a target assigned a local ID *p* in camera *i* has appeared in other cameras *j* (j∈1,2,…,M,j≠i), we calculate $D(S_{i,p},S_{j,q})$—the distance between $S_{i,p}$ and $S_{j,q}$ ($S_{j,q}\in S_{j}$)—by using Equation (9), which will be added to a tracklet similarity matrix $R_{i,j}$.
**Algorithm 2:** Calculation of Similarity List *R* and Generation of a Global ID List $G_{i}$**Input** **:**    Vector sequence set $S=\{S_{1},S_{2},\ldots ,S_{i},\ldots ,S_{M}\}$**Output** **:**    Similar local ID set $G_{i}=\left\{G_{i,1},G_{i,2},\ldots ,G_{i,N}\right\}$1:Generate empty ranking list *R*2:**for all**$S_{i}\left(i=1,2,\ldots ,M-1\right)\in S$**do**3: **for all** $S_{i,p}\in S_{i}$ **do**4:  **for all** $S_{j}\left(j=i+1,i+2,\ldots ,M\right)\in S$ **do**5:   **for all** $S_{j,q}\in S_{c}$ **do**6:    Calculate DTW distance $D(S_{i,p},S_{j,q})$ using Equation (9)7:    Set the element of row *p* and column *q* in $A_{i,j}$ to $D(S_{i,p},S_{j,q})$, i.e., $A_{i,j}\left(p,q\right)=D\left(S_{i,p},S_{j,q}\right)$8:   **end for**9:  **end for**10: **end for**11: Calculate matching matrix $A_{i,j}$ according to Equation (10)12: Add all $A_{i,j}\left(p,q\right)=1$ to $G_{i}$ in the form of a tuple (p,q).13:**end for**

Given $R_{i,j}$, we calculate a matching matrix $A_{i,j}\in {0,1}^{N\times N}$, where each element $A_{i,j}\left(p,q\right)$ denotes a binary value of 1 or 0—which represents matching (i.e., $A_{i,j}\left(p,q\right)=1$) and non-matching (i.e., $A_{i,j}\left(p,q\right)=0$) operations. If tracklets *p* and *q* have a high similarity score $D(S_{i,p},S_{j,q})\to 1$, we should have $A\left(p,:\right)A{\left(q,:\right)}^{T}=1$. On the contrary, for a small similarity score, $A\left(p,:\right)A{\left(q,:\right)}^{T}=0$, which implies that *p* and *q* are not the same target. This bipartite-graph-matching problem can be solved by the following optimization problem:

(10) $$\begin{array}{c} A_{i,j}^{*}=\arg\max_{G_{i,j}}\left|\right|R_{i,j}⊙G_{i,j}{\left|\right|}_{2},\\ s.t.\forall p,\sum G(p,:)\leq 1,\\ \forall q,\sum G(:,q)\leq 1, \end{array}$$

where ⊙ denotes element-wise matrix multiplication, and $\left|\right|\cdot {\left|\right|}_{2}$ denotes the L2 norm of the input matrix. The constraints ensure the mutual exclusion of trajectories such that each detection occupies using at most one trajectory. Based on this, the optimization problem can be efficiently solved by the Hungarian algorithm [30].

Finally, given the sets $G=\{G_{1},G_{2},\ldots ,G_{M}\}$ containing the local tracklet matching information, we assign the global ID to each local matching information and create a set $I^{G}$ for *V*. $I^{G}$ has the same configuration as $I^{L}$ except that the ID is global rather than local, and the coordinates possess the average coordinates of local IDs mapped to the global IDs. We assign a new global ID to the elements with the tuple (p,:) and/or (:,q) for $\forall \left(p,q\right)\in G_{i}$ ($\forall G_{i}\in G$). The overall pipeline is described in Algorithm 3.
**Algorithm 3:** Overall Pipeline to Generate Global Information**Input** **:**    Video set $V=\{V_{1},V_{2},\ldots .,V_{M}\}$ collected from *M* cameras**Output** **:**    Global video information $I^{G}$1:**for all**$V_{i}\in V$**do**2: Generate a local video information $I_{i}^{L}$ using a multi-object tracker [31]3:**end for**4:**for all** local video information $I_{i}^{L}\in I^{L}$ **do**5: Calculate the projected footprint coordinate ${\left(x,y\right)}^{P}$ using homography matrix *H* and generate a projected video information $I^{P}$6:**end for**7:**for all**$I_{i}^{P}\in I^{P}$**do**8: **for all** *p* in $V_{i}$ **do**9:  Generate local tracket $T_{i,p}$ and create vector sequence set $S_{i,p}$ using Algorithm 1 ($T_{i,p}\in T_{i},S_{i,p}\in S_{i}$)10: **end for**11:**end for**12:Calculate similarity using DTW and generate similar local ID list set $G=\{G_{1},G_{2},\ldots ,G_{M}\}$ using Algorithm 213:For $\forall \left(p,q\right)\in G_{i}$ ($\forall G_{i}\in G$), assign a new global ID to the elements with the tuple (p,:) and/or (:,q)14:Create an empty global video information $I^{G}$15:**while** current frame < the end of frame **do**16: In $I^{P}$, find all existing ID on the current frame and update to global ID, calculate average coordinates17: Add the update to $I^{G}$18:**end while**

## 4. Performance Evaluation

### 4.1. Experimental Setup

**Figure 4:**
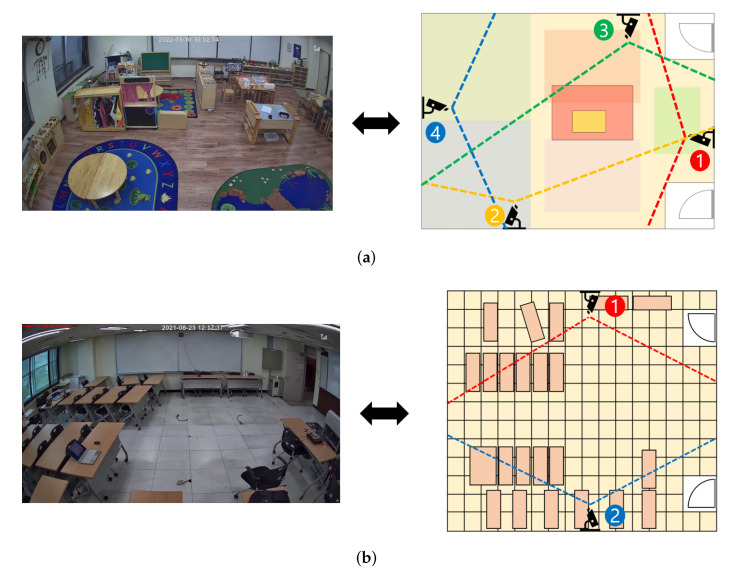
The testbeds and projected maps at two different locations (**a**) with four cameras and (**b**) two cameras, respectively.

To evaluate our method, we have conducted experiments using Wisenet’s four-channel camera systems, SNK-B73047BW [48]. For experiments, we constructed overlapping FOV environments by installing up to four cameras in various places including classrooms and daycare centers, as shown in Figure 4. Each video had three different configurations: 180,000, 135,000, and 90,000 frames.

In the first stage of MTMCT, wherein the Ground Projector generates the sets of local tracklet (Section 3.3), we consider different parameters for the KLT algorithm-based frame-skipping method with a fixed sampling interval, namely *Skip value*: 1 (no skip), 5, and 10. A larger skip value reduces the number of frames used for target tracking and speeds up the calculation. The frames between the two selected frames are inferred by applying the KLT tracker. To evaluate its effect and find the desired skip parameter, we compared the performance for the same dataset with different skip parameters. Moreover, as explained in Section 3.3, we considered the moving direction of the targets to filter out the errors in tracklet positions caused by camera distortion.

In the second step, we performed global ID matching based on the similarity analysis between projected tracklets using the DTW algorithm and measured accuracy. For a given coordinate of each target, three different features—(i) scalar, (ii) vector, and (iii) unit vector based on Equations (6)–(8), respectively—were used to generate input sequences for DTW similarity analysis, and we compare their effect on the accuracy of global ID matching.

### 4.2. Evaluation Results

**Figure 5:**
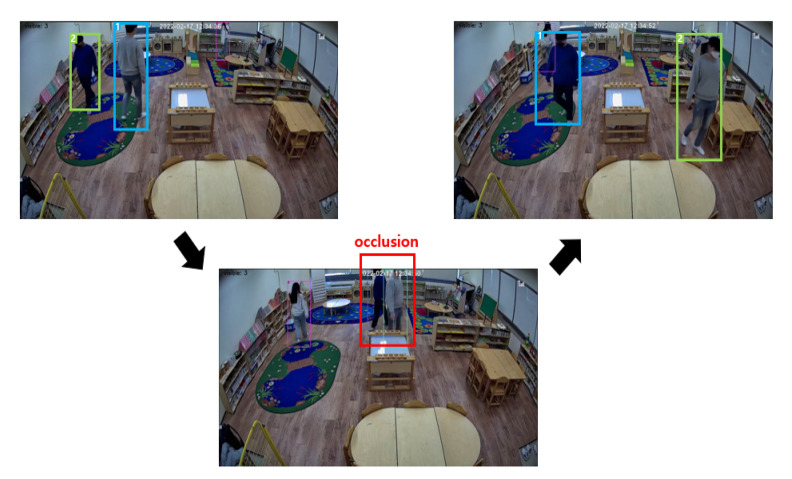
ID switching owing to short-term occlusion.

The accuracy of the proposed global ID-matching method mainly relies on the tracklet data obtained from the output of the Multi-Object Tracker. This implies that the performance of the Multi-Object Tracker used has a significant impact. In particular, it is sensitive to the problems of ID switching and fragmentation. ID switching is a phenomenon in which an existing ID assigned to object X is incorrectly assigned to another object Y. It can be caused by several reasons, including occlusion, where other objects partially or totally obscure the identified object during a short period. For instance, Figure 5 shows a case of ID switching due to occlusion when two targets assigned with IDs 1 and 2 intersect for a short time.

In order to evaluate the performance of our proposal, therefore, it is important to understand the basic characteristics of the object tracker and obtain a ground-truth dataset for performance evaluation. To this end, we collected ground-truth values by preprocessing the experimental image data to identify information on these ID-switching problems and measured the number of incorrect ID switching occurrences.

First, we examine the effect of the Frame Skipping method applied to alleviate the fundamental accuracy problem of the MOT.

Table 1 lists the number of incorrect ID switching for each camera in a four-camera network with three different skip values. As ID switching occurs depending on the state of the video image, it varies depending on the installation location or angle of the camera. In our experiment, Re-ID failure occurred most frequently in camera 2, i.e., 48 out of a total of 150 data. This can be attributed to the fact that there are no obstacles near the wall where camera 2 was installed; thus, the target can move under camera 2. Only the upper body was recognized, which reduces the accuracy of Re-ID.

**Figure 6:**
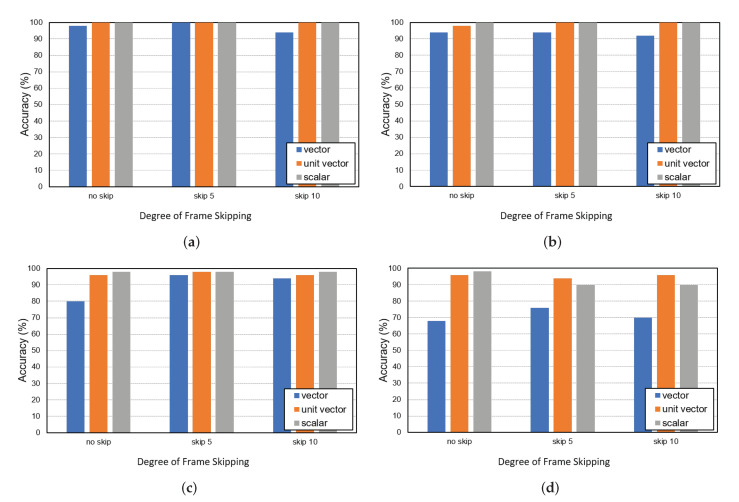
Global ID matching results for three targets in several overlapping FOV indoor scenarios with two cameras: (**a**) cameras #1 and #4, (**b**) cameras #1 and #3, (**c**) cameras #1 and #2, and (**d**) cameras #2 and #3.

Figure 6 shows the global ID-matching accuracy for three target persons in several FOV indoor environments covered by two cameras with three different target’s movement features (i.e., scalar, vector, and unit vector) and three different skip values (i.e., no-skip, skip 5, and skip 10). Table 2 lists their F1 scores. In calculating the accuracy of the assignment results, an ID assignment is only considered successful if all global ID-matching results are correctly assigned to all three targets across the cameras. As shown in Figure 4, the overlapping area of cameras 1 and 4 was relatively wide, and camera 1 was deployed to observe the obstacles around the window easily. Thus, the results of experiments using cameras 1 and 4 and camera 1 and 3 in Figure 6a,b show very high accuracy for all the three motion features.

As shown in Figure 6c,d, the results including camera 2 exhibit relatively low performance. We analyzed the cause of matching failure and observed that there were mainly two reasons. First, the bounding box of the Multi-Object Tracker itself fluctuated severely in the videos from camera 2, and it had a greater impact on the similarity analysis between sequences obtained from vector motion features using direction information. Next, we observed high Re-ID errors in videos from camera 2 due to its underlying deployment environment. Table 3 shows the Re-ID failure rate for the results in Figure 6d for cameras 2 and 3, which yielded the lowest performance among the four combinations. In the cases of original (no skip) and sampling with a skip value of 5 (skip 5), global ID-matching failure due to Re-ID failure accounts for more than half of all the failures. This implies that most errors occur not because of the proposed matching solution but due to the inherent Re-ID problem of Multi Object Tracker. After all, the Multi-Object Tracker’s Re-ID problem (which is outside the scope of our paper) greatly affects the performance of the proposed technique.

In the case of the combination of cameras 1 and 2 it can be seen that using frame skipping with a skip value of 5 (skip 5) significantly improved the performance of the unit vector feature, although the accuracy degrades again when using a higher skip value, i.e., 10. This is because ‘skip 10’ predicts the target’s position over a longer frame, resulting in a loss of original information. Based on these results, we can observe the effect on the performance of the frame-skipping technique based on the KLT. That is, despite a small amount of frames used to generate sequence sets compared to the original data, the frame-skipping scheme improved the matching accuracy.

**Figure 7:**
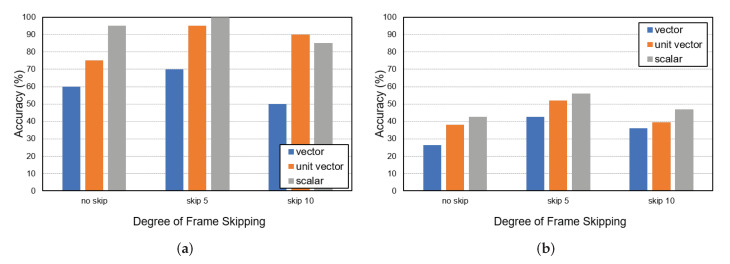
Global ID-matching accuracy with two cameras for (**a**) five persons and (**b**) ten persons.

Next, we conducted experiments for additional people in the overlapping FOV indoor environment with two cameras. Figure 7 shows the experimental results for five and ten persons with three different frame skipping and motion features. Table 4 lists their F1 scores. In the five-person experiment, the proposed scheme achieved an accuracy of 100% with a frame skipping value of 5 and scalar motion feature. In the case of the ten-person experiments, the matching was counted as a failure even in one false matching among 10 matchings, so a significant decrease in matching accuracy was observed. Note that the ten-person experiment is a very congested scenario. Nevertheless, we observed that frame skipping improved the performance for all motion features in these experiments. When the vector feature was used, the accuracy was shown to be lower than the other two features. This is because the vector takes into account the stride length and directionality of the target. As previously explained, accurate stride measurements are not possible for reasons such as undulations in the bounding box or errors in the ground projection. Therefore, it can be confirmed that the scalar and the unit vector are suitable motion features for the proposed method.

Indoor environment have blind spots due to obstacles and other reasons, which decrease MOT accuracy. Therefore, it is necessary to intentionally construct an overlapping FOV environment using redundant cameras. In this regard, in the following experiment, we conducted measurements with more cameras to test whether our global ID-matching method will show stable performance for using two or more cameras.

**Figure 8:**
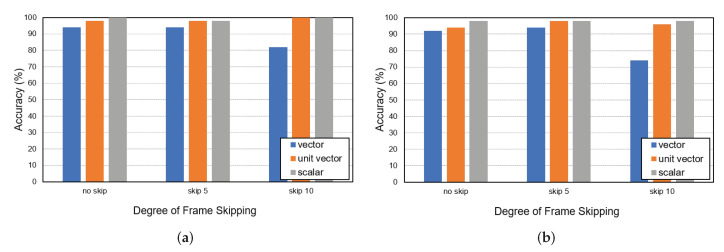
Global ID-matching accuracy with (**a**) 3 cameras (cameras 1, 2, and 3), and (**b**) 4 cameras.

Figure 8a,b show high accuracy using three and four cameras, respectively, for three persons. While calculating accuracy, it was considered a success only if the targets of all the cameras matched correctly. Despite the increase in the number of cameras, the proposed technology achieved high accuracy. It achieved the best performance with the skip values of 5 and 10 for vector and both scalar and unit vector features, respectively. We investigated and summarized the errors caused by Re-ID of Muilti Object Tracker among the causes of the failure of matching for four cameras in Table 5. The results indicate that most errors (in the experiments with the unit vector and scalar features) are not related to our algorithms but rather are due to the inherent noise involved in the sequence sets caused by Re-ID problems.

Finally, we studied the impact of our approach in reducing the effects of computational complexity on matching accuracy. As explained in Section 3.2, sequence sets were generated using Algorithm 1 and KLT-based frame skipping. In addition, we further reduced the amount of data required to generate sequence sets by selectively using frames from each video $V_{i}$. Given $V_{i}$ with the default frame rate of 24 frames/sec, we use interval sampling (also known as systematic sampling), which selects every *k*-th frame in the video, where we have tested for k= 1 (default/original), 2 (half frame rate), and 3 (one-third), which correspond to 24, 12, and 8 frames/sec, respectively.

**Figure 9:**
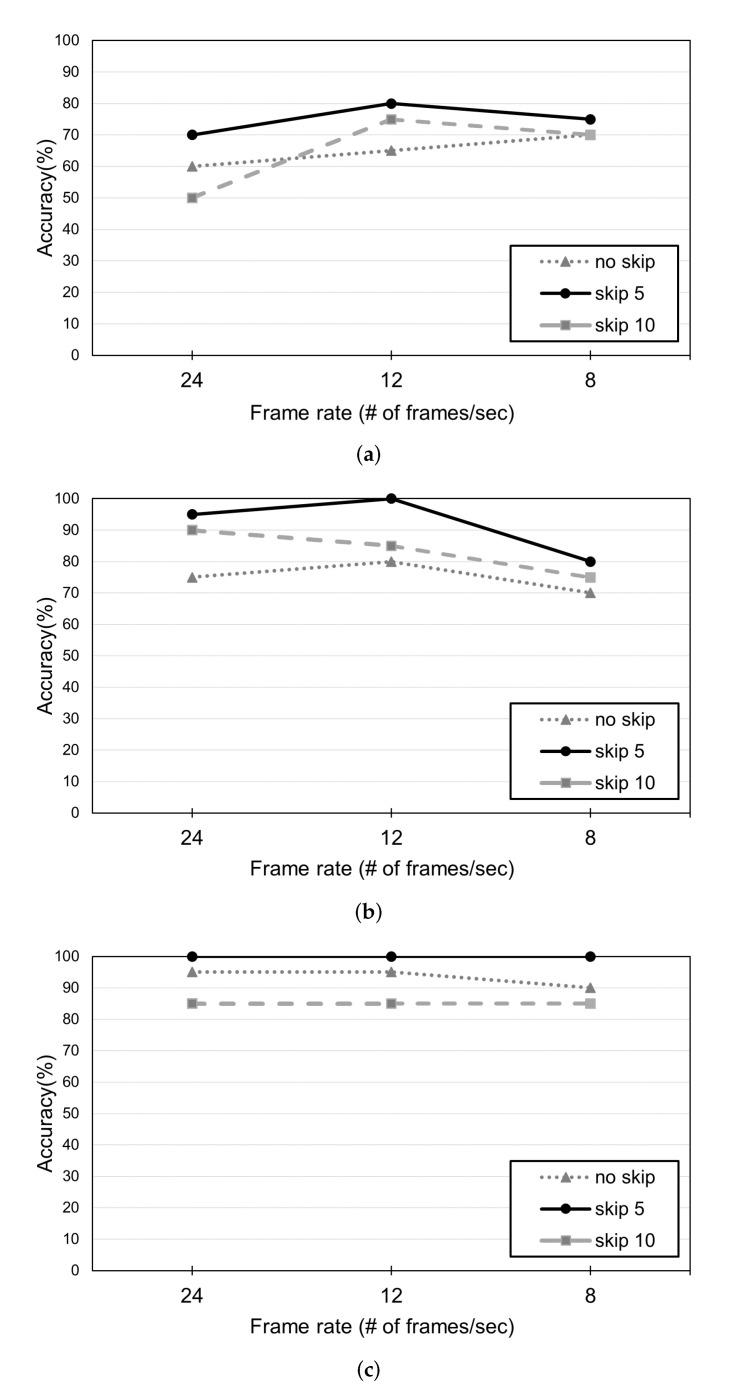
Effect of frame sampling rate for three movement features: (**a**) vector, (**b**) unit vector and (**c**) scalar.

Figure 9 shows the experimental performance of two cameras and five persons considering vector, unit vector, and scalar features. In the results, we have observed a clear effect on performance with frame rate for all the three movement features and a significant performance improvement using the half rate of 12 frames/s instead of using the original frame rate. If the frame rate is too low (e.g., 8 frames/s), the accuracy will decrease again. While using unit vectors for generating sequence sets, the original video without frame skipping at 24 frames/s had an accuracy of 90%. On the other hand, it is improved to an accuracy of 100% when applying frame skipping with a skip of 5 at a half frame rate, i.e., 12 frames/s, where the amount of data corresponds to 1/10 of the original data. In the case of scalar feature, sampling with a skip value of 5 achieved an accuracy of 100%, and there was no performance degradation even when the amount of information is reduced by 1/3 with 8 frames/s. These results imply that the proposed approach showed high accuracy even with a smaller amounts of data (1/10 of that of the original data), lightweight computational overhead for DTW similarity, and matching tasks.

## 5. Conclusions

In this paper, we proposed a new lightweight matching method for MTMC tracking consisting of two steps: (i) extracting targets’ motion information based on a ground projection method, and (ii) matching the tracklets using similarity analysis based on the Dynamic Time Warping (DTW) algorithm. We reduced the computational overhead by leveraging a KLT-based frame skipping and smoothing method to reduce computational costs in using targets’ location information to generate input sequence sets for our matching algorithm. In addition, three different location features including scalar, vector, and unit vector have also been studied to derive the best input feature for similarity analysis to improve matching accuracy. Extensive experiments demonstrated the effectiveness of the proposed method, showing that our scheme achieved high accuracy in most overlapping FOV environments. In dense environments, performance degradation occurred, but it has been shown that many errors occur not because of the proposed matching solution but due to the inherent Re-ID problem of the Multi Object Tracker.

**Limitation and Future Work.** A significant limitation is that the accuracy of the proposed framework strongly relies on the performance of the Multi-Object Tracker, since the tracklet data are obtained from the output of the Multi-Object Tracker. Although we alleviate the inborn position error caused by camera distortion by exploiting the moving direction information of the target instead of the absolute position of the target, the matching based on the similarity analysis suffers from the errors even with the proposed features when the camera distortion is severe.

In the future, we would like to extend our work in the following three categories: (i) employing adaptive feature selection for different object’s movement features, (ii) optimizing tracklet matching using graph models and/or Bayesian formulation, and (iii) reducing the computational complexity of DTW for obtaining similarity measures between feature sequences by applying fast DTW algorithms.

## Figures and Tables

**Table 1:** Incorrect ID switching in a four-camera network with three different skip values.

Camera ID	Camera 1	Camera 2	Camera 3	Camera 4
Original	5	21	2	7
Skip 5	2	13	3	5
Skip 10	11	14	2	8
Total	18	48	7	20

**Table 2:** F1 score for global tracklet matching in cameras 2 and 3 FOV environments.

cameras #1 and #4	Original	Skip 5	Skip 10
Vector	0.98	1.0	0.94
Unit vector	1.0	1.0	1.0
Scalar	1.0	1.0	1.0
**cameras #1 and #3**	**Original**	**Skip 5**	**Skip 10**
Vector	0.96	0.96	0.9
Unit vector	0.98	1.0	1.0
Scalar	1.0	1.0	1.0
**cameras #1 and #2**	**Original**	**Skip 5**	**Skip 10**
Vector	0.96	0.98	0.87
Unit vector	0.98	0.98	0.98
Scalar	1.0	0.98	0.98
**cameras #2 and #3**	**Original**	**Skip 5**	**Skip 10**
Vector	0.77	0.83	0.80
Unit vector	0.98	0.97	0.98
Scalar	0.98	0.93	0.94

**Table 3:** Ratio of errors due to (inherent) Re-ID to the total number of failures for global tracklet matching in cameras 2 and 3 FOV environments.

Feature	Original	Skip 5	Skip 10
Vector	10/16	6/12	5/15
Unit vector	2/2	3/3	1/2
Scalar	1/1	4/5	2/5

**Table 4:** F1 score for Global Tracklet Matching with Two Cameras for Five Persons and Ten Persons.

cameras #1 and #4	Original	Skip 5	Skip 10
Vector	0.80	0.85	0.77
Unit vector	0.88	0.98	0.96
Scalar	0.98	1.0	0.96
**cameras #1 and #3**	**Original**	**Skip 5**	**Skip 10**
Vector	0.36	0.56	0.50
Unit vector	0.48	0.65	0.56
Scalar	0.52	0.68	0.61

**Table 5:** Ratio of errors due to Re-ID to the total number of failures for global tracklet matching in the experiment with four cameras.

Feature	Original	Skip 5	Skip 10
Vector	2/4	3/3	7/13
Unit vector	3/3	1/1	1/2
Scalar	1/1	1/1	0/1
